# Kinase Suppressor of Ras 1 Is Not Required for the Generation of Regulatory and Memory T Cells

**DOI:** 10.1371/journal.pone.0057137

**Published:** 2013-02-19

**Authors:** Marie Le Borgne, Erin L. Filbert, Andrey S. Shaw

**Affiliations:** 1 Department of Pathology and Immunology, Washington University School of Medicine, St. Louis, Missouri, United States of America; 2 Howard Hughes Medical Institute, Washington University School of Medicine, St. Louis, Missouri, United States of America; Beth Israel Deaconess Medical Center, Harvard Medical School, United States of America

## Abstract

The mammalian target of rapamycin (mTOR) kinase is a critical regulator of the differentiation of helper and regulatory CD4+ T cells, as well as memory CD8+ T cells. In this study, we investigated the role of the ERK signaling pathway in regulating mTOR activation in T cells. We showed that activation of ERK following TCR engagement is required for sustained mTOR complex 1 (mTORC1) activation. Absence of kinase suppressor of Ras 1 (KSR1), a scaffold protein of the ERK signaling pathway, or inhibition of ERK resulted in decreased mTORC1 activity following T cell activation. However, KSR1-deficient mice displayed normal regulatory CD4+ T cell development, as well as normal memory CD8+ T cell responses to LCMV and *Listeria monocytogenes* infection. These data indicate that despite its role in mTORC1 activation, KSR1 is not required *in vivo* for mTOR-dependent T cell differentiation.

## Introduction

The differentiation of naïve T cells into specialized effector, memory, and regulatory T cells, is critical for mounting an appropriate immune response to pathogens, and tumors as well as for maintaining tolerance to self. The mammalian target of rapamycin (mTOR) is a conserved serine/threonine kinase that has been implicated in many of these events. mTOR plays a role in peripheral tolerance, as mTOR inhibition during T cell activation can lead to anergy [1], as well as promote the differentiation of CD4+ T cells into regulatory T cells (Tregs) [2]–[4]. In contrast, mTOR signaling is required for the differentiation of naïve CD4+ T cells into Th1, Th2 and Th17 effector T cells [3], [5], [6]. Finally, inhibition of mTOR promotes the differentiation of memory CD8+ T cells, as treatment of mice with the mTOR inhibitor rapamycin during infections with viruses and bacteria improves the generation and maintenance of pathogen-specific memory CD8+ T cells [7]–[9].

mTOR is present in two different complexes called mTOR complex 1 (mTORC1) and mTOR complex 2 (mTORC2). Each complex contains a distinct scaffold protein such as regulatory-associated protein of mTOR (Raptor) and rapamycin-insensitive companion of mTOR (Rictor) in mTORC1 and mTORC2, respectively [10]. The requirement for mTORC1 versus mTORC2 in T cells varies with cell type. For example, mTORC1 is required for Th17 and CD8+ memory T cell differentiation, whereas mTORC2 is required for Th2 differentiation [3], [6]–[8]. Inhibition of both mTORC1 and mTORC2 is required for the enhanced generation of Tregs [3]. In T cells, as in other eukaryotic cells, mTOR is activated in response to environmental cues, such as growth factors, and metabolic cues, such as nutrients. However, mTOR is also activated in T cells in response to signals such as TCR engagement, co-stimulation, and cytokines [10].

Two main pathways have been described to regulate mTORC1 activation in T cells, namely the AMP-activated protein kinase (AMPK) pathway and the phosphoinositide 3-kinase (PI3K)-AKT pathway [9]–[11]. The response to both signaling pathways are integrated by the tuberous sclerosis 1 (TSC1) – tuberous sclerosis 2 (TSC2) complex. When active, the TSC1/TSC2 complex acts as a GTPase activating protein (GAP) for Rheb, a crucial activator of mTORC1. Inactivation of Rheb by the TSC1/2 complex therefore inhibits mTORC1 signaling. Phosphorylation of TSC2 by AKT inhibits its GAP activity, whereas phosphorylation of TSC2 by AMPK stimulates it [12]. This explains how AKT activates and AMPK inactivates mTORC1.

In other cell types, additional pathways, such as the ERK/mitogen-activated protein kinase (MAPK) pathway, have been shown to regulate mTORC1 activation [13]–[15]. ERK is activated in response to growth factors and can directly phosphorylate TSC2, disrupting the TSC1/TSC2 complex, thereby increasing mTOR activity [13]. ERK can also phosphorylate p90 ribosomal protein S6 kinase (RSK), which phosphorylates TSC2 [15]. While it is known that during T cell activation, ERK is activated and recruited to the immunological synapse [16], [17], it is unknown if ERK regulates mTORC1 in T cells.

Previous work in our laboratory showed that kinase suppressor of Ras 1 (KSR1), a scaffold protein for Raf, MEK and ERK [18], plays a critical role in the optimal activation of ERK in T cells [16], [17], [19]. Furthermore, KSR1 is known to associate with mTOR, Raptor and Rictor in cycling 293T cells [20]. Thus, KSR1 might regulate mTOR activation in T cells, both by controlling ERK activation and by bringing together members of the ERK and the mTOR pathway.

Here we examined if ERK and KSR1 play a role in mTORC1 activation in T cells. We showed that mTORC1 activation was decreased in the presence of a MAPK inhibitor and in KSR1-deficient T cells during T cell activation. However, KSR1-deficiency did not affect the development of regulatory T cells or CD8+ memory T cells *in vivo*. Therefore, although KSR1 is required for optimal mTORC1 activation in T cells, it is not necessary for mTORC1-dependent T cell development and function *in vivo*.

## Results

### TCR-induced mTORC1 activation depends on the ERK signaling pathway

ERK was previously shown to activate mTORC1 in a kidney embryonic stem cell line through the phosphorylation RSK and the direct phosphorylation of TSC2 [13]–[15]. As ERK is activated following TCR engagement in T cells, we postulated that it might also play a role in TCR-induced mTOR activation. To test this, we measured the level of phosphorylation of the ribosomal protein S6 kinase (S6K), a target of mTORC1, induced by TCR stimulation in the presence or absence of the MEK inhibitor UO126. Mouse splenocytes were treated for 1 hour with UO126 and then activated with soluble anti-CD3 and anti-CD28 antibodies. Two to 48 hours after stimulation, we analyzed the phosphorylation of ERK and of S6K on different sites in T cells by flow cytometry. S6K can be directly phosphorylated on T389 by mTORC1 [21], [22], whereas phosphorylation on T421/S424 may be mediated through the MAPK pathway independently of mTOR in immune cells [23].

**Figure 1:**
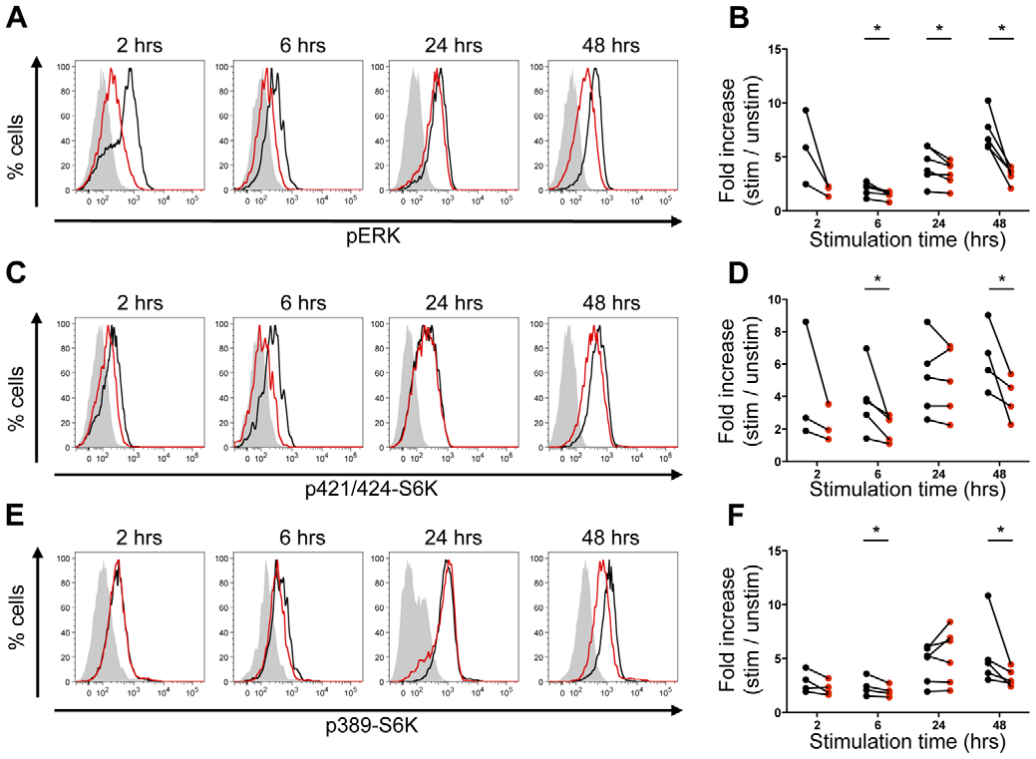
MEK inhibition decreases mTOR activity in T cells. Splenocytes from B6 mice were pretreated or not for one hour with 10 µM UO126 and cultured for 2 to 48 hrs with or without anti-CD3 + anti-CD28 stimulation (5 µg/mL each). Phosphorylation of ERK (A, B), T421/S424-S6K (C, D) and T389-S6K (E, F) in CD8+ T cells was measured by flow cytometry. A, C, E: Representative histograms of unstimulated cells (filled histogram), cells stimulated with CD3+CD28 and treated (red line) or not (black line) with UO126, gated on CD8+ T cells. Graphs are representative of 3 to 5 experiments. C, D, F: Fold increase phosphorylation of ERK, T421/S424-S6K and T389-S6K in anti-CD3 + anti-CD28 stimulated CD8+ T cells treated (red) or not (black) with UO126, compared to unstimulated cells. Each dot represents an independent experiment. p-ERK, p-T421/S424-S6K and p-T389-S6K levels were normalized within each experiment to unstimulated control T cells. *: p<0.05, paired t-test.

Stimulation with anti-CD3 + anti-CD28 induced ERK phosphorylation within 2 hrs. After 6 hrs, ERK phosphorylation levels decreased, before being up-regulated again after 24 and 48 hrs of stimulation (Figure 1A and 1B). As expected, ERK phosphorylation was decreased in CD8+ T cells treated with UO126. Phosphorylation of S6K on T421/S424 was also decreased (Figure 1C and 1D); this might be independent of mTORC1 and result directly from ERK inhibition [23]. Finally, anti-CD3 + anti-CD28 stimulation induced S6K phosphorylation on T389, a direct target of mTORC1, which could be detected after 2 hrs of stimulation, and peaked after 24 to 48 hrs (Figure 1E and 1D). Treatment with UO126 induced a 40% decrease in phosphorylation of S6K on T389 48 hours after stimulation (Figure 1E and 1F), but no significant difference at earlier time points. We observed the same effect in UO126-treated CD4+ T cells (data not shown). We confirmed by performing western blots that phosphorylation of ERK and S6K at T389 was decreased in UO126-treated T cells after 48 hrs of stimulation (Supplementary Figure S1). This suggests that ERK signaling is required for sustained activation of mTORC1 in T cells.

### KSR1 is required for sustained mTORC1 activation following T cell stimulation

**Figure 2:**
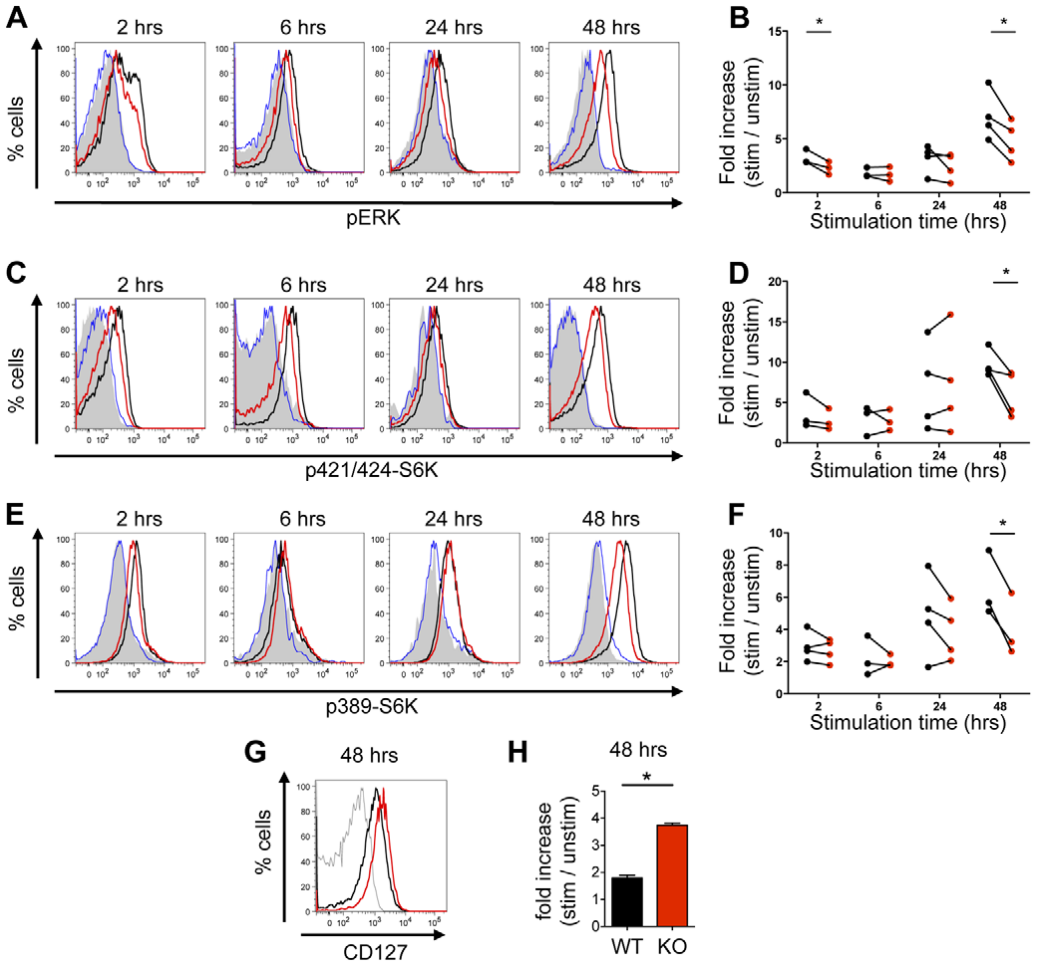
mTOR activity is decreased in KSR1-deficient T cells. Splenocytes from WT or KSR1-/- mice were cultured with or without anti-CD3 + anti-CD28 (5 µg/mL each) stimulation for 2 to 48 hrs. Phosphorylation of ERK (A, B), T421/S424-S6K (C, D) and T389-S6K (E, F), and expression of CD127 (G,H) in CD8+ T cells was measured by flow cytometry. A, C, E: Representative histograms of unstimulated WT cells (filled grey histogram), unstimulated KO cells (blue line), and WT (black line) or KO (red line) cells stimulated with CD3+CD28, gated on CD8+ T cells. Graphs are representative of 3 to 4 experiments. C, D, F: Fold increase phosphorylation of ERK, T421/S424-S6K and T389-S6K in stimulated WT (black) and KO (red) CD8+ T cells compared to non-stimulated cells. Each dot represents an independent experiment. p-ERK, p-T421/S424-S6K and p-T389-S6K levels were normalized within each experiment to unstimulated control T cells. *: p<0.05, paired t-test. G-H. Representative histograms (G) and fold increase in comparison to non-stimulated control T cells (H) of CD127 expression in WT (black) and KO (red) cells stimulated with CD3+CD28, gated on CD8+ T cells. Grey line: isotype control. H. Data is presented as mean + SEM and is representative of two independent experiments. *: p<0.05, unpaired t-test.

As MEK inhibition affected T cell proliferation 48 hours after stimulation, we wanted to confirm whether the decreased mTORC1 activity in UO126-treated T cells was due to an impaired activation of mTORC1 by ERK, and not to some indirect effect of decreased proliferation. MEK and ERK activation are impaired during T cell activation in cells deficient for the scaffold molecule KSR1 [17]. However, contrary to previous studies [17], no defect in T cell proliferation was seen (data not shown); this difference might stem from differences in the genetic background of the mice used in these experiments. We therefore used KSR1-deficient T cells to confirm that the ERK signaling pathway activates the mTOR pathway following T cell stimulation, by looking at S6K phosphorylation in T cells stimulated with anti-CD3 and anti-CD28. As expected, we observed that ERK phosphorylation was decreased in KSR1-deficient T cells (Figure 2A and 2B). The effect was more pronounced 2 and 48 hours after stimulation (30% and 33% decreased ERK phosphorylation in KSR1-deficient CD8+ T cells, respectively). S6K phosphorylation at T421/S424 and T389 was normal in KSR1-deficient T cells up to 24 hours after stimulation, but was decreased at 48 hours both in CD8+ T cells (38% decrease for T421/S424 and 40% decrease for T389, Figure 2C–2F) and in CD4+ T cells (33% decrease for T421/S424 and 29% decrease for T389, data not shown). Western blots confirmed the decreased phosphorylation of ERK and of S6K at T389 in KSR1-deficient T cells stimulated for 48 hrs (Supplementary Figure S1). These data demonstrate that KSR1 is required for sustained activation of mTORC1 during T cell activation, and confirm that ERK signaling pathway is necessary for optimal mTORC1 activation.

As mTORC1 inhibition with rapamycin has been shown to favor the generation of CD8+ memory T cell precursors *in vitro* [9], we assessed if KSR1-deficiency led to a similar phenotype. We assessed the expression of different markers associated with memory precursor CD8+ T cells, namely CD62L (important for lymph node homing), CD69 (lymph node retention), and CD127 (IL-7Rα, essential for the maintenance of CD8+ memory T cells [24]), in wild-type and KSR1-deficient CD8+ T cells stimulated for 48 hrs with anti-CD3 and anti-CD28 *in vitro*. We did not see any difference in CD62L and CD69 expression (data not shown), but observed increased CD127 expression in CD8+ KSR1-/- T cells (Figure 2G–2H). Therefore, these data indicate that the decreased mTORC1 activation in KSR1-deficient CD8+ T cells is associated with the increased generation of CD8+ T cells with some characteristics of memory precursors.

### KSR1 deficiency does not affect the development of regulatory T cells in vivo

**Figure 3:**
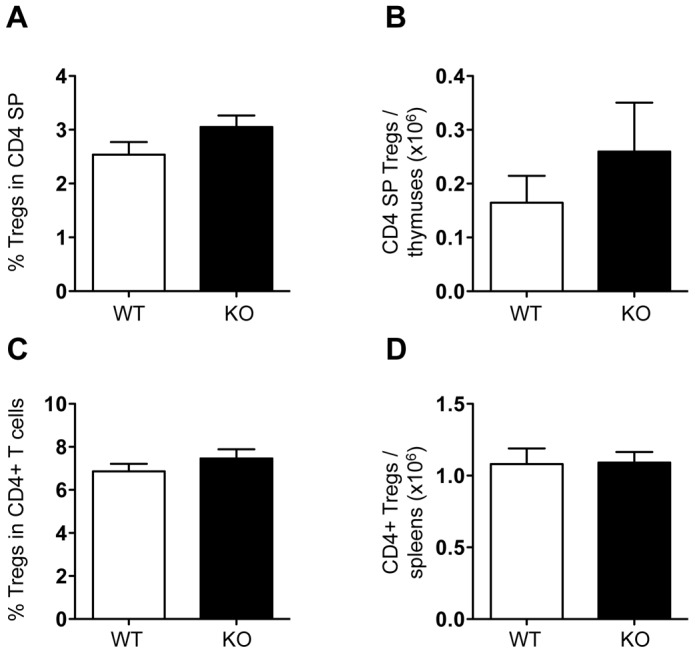
Regulatory CD4+ T cell development is normal in KSR1-deficient T cells. Thymocytes (A, B) and splenocytes (C, D) of WT (white bars) and KSR1-/- (black bars) mice were analyzed for the presence of regulatory CD4+ T cells by flow cytometry. A, C: Percentage of FoxP3+ CD25+ cells in CD4+ CD8- single positive (SP) thymocytes (A) and CD4+ CD3+ T cells (B). B, D: Numbers of FoxP3+ CD25+ CD4 SP by thymuses (B) and CD4+ T cells by spleens (D). Data is presented as mean + SEM and represent 10-12 mice per group. There were no significant differences between WT and KO mice (t-test).

As mTOR activity has been shown to control T cell differentiation in regulatory, effector and memory T cells, we wanted to assess if the decreased mTORC1 activity in KSR1-deficient T cells has any effect on T cell differentiation. It was previously reported that mTOR deficiency or inhibition of mTORC1 with rapamycin enhances the differentiation of CD4+ T cells into FoxP3+ Tregs [3], [4], [25]. As mTORC1 activity was decreased in KSR1-deficient CD4+ T cells, we tested if the development of Tregs was increased in KSR1-/- mice. We did not observe any significant difference in the percentage or numbers of FoxP3+ CD4+ Tregs in the thymus (Figure 3A and 3B) or spleen (Figure 3C and 3D) between wild-type and KSR1-/- mice. Therefore, the decreased mTOR activity following TCR engagement in KSR1-deficient CD4+ T cells does not lead to increased Treg development.

### KSR1 deficiency does not affect the generation of memory CD8+ T cells in vivo

**Figure 4:**
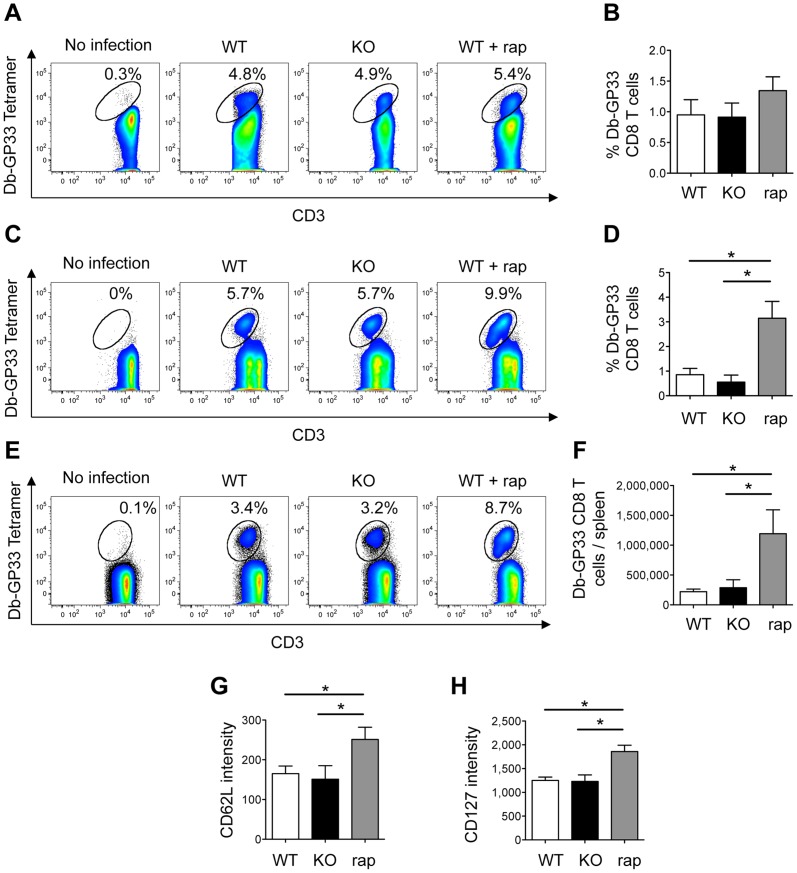
Normal effector and memory CD8+ T cell response to LCMV in KSR1-deficient mice. WT, KSR1-/-, and WT mice treated with rapamycin were infected with 10^5^ pfu of LCMV-Armstrong, and presence of GP33 epitope specific CD8+ T cells was analyzed by flow cytometry 7 (A, B) and 30 (C–H) days after infection in the blood (A–D) and spleen (E–H). A, C, E: Percentage of Db-GP33 Tetramer positive cells in CD8+ T cells. Plots are representative of two experiments with 5 mice per group. B, D: Percentage of Db-GP33 Tetramer+ CD8+ T cells in peripheral blood mononuclear cells. F: Number of Db-GP33 Tetramer+ CD8+ T cells per spleen 30 days after infection. G, H: CD62L and CD127 expression in Db-GP33 Tetramer+ CD8+ T splenocytes 30 days after infection. Data is presented as mean + SEM and is representative of two experiments with 5 mice per group. *: p<0.05, t-test.

Inhibition of mTORC1 with rapamycin has also been shown to increase memory CD8+ T cell differentiation in several infection models [7], [8]. Therefore, we hypothesized that the decreased mTORC1 activity in activated KSR1-deficient CD8+ T cells might lead to increased memory CD8+ T cell differentiation. To test this, we first looked at the generation of virus-specific CD8+ memory T cells in KSR1-/- mice during the course of an acute lymphocytic choriomeningitis virus (LCMV) infection. As a positive control, we treated a cohort of wild-type mice with rapamycin starting from the day before infection until the end of the experiment, as this has been shown to increase memory T cell generation in this model [7]. Rapamycin treatment or KSR1-deficiency did not affect the generation of LCMV-specific effector T cells (Figure 4A and 4B). However, rapamycin treatment increased the number of LCMV-specific memory T cells 30 days post-infection in the blood and the spleen of infected mice (Figure 4C–4F). Furthermore, the functional quality of the memory T cells was improved, with increased CD62L [26], Figure 4G) and CD127 expression ([24], Figure 4H). These data confirmed that mTORC1 inhibition increases the generation and the quality of LCMV-specific memory CD8+ T cells *in vivo*, as previously described [7]. However, we could not detect any difference in the number and quality of the LCMV-specific memory CD8+ T cells in KSR1-/- mice 30 days after infection (Figure 4C–4H).

**Figure 5:**
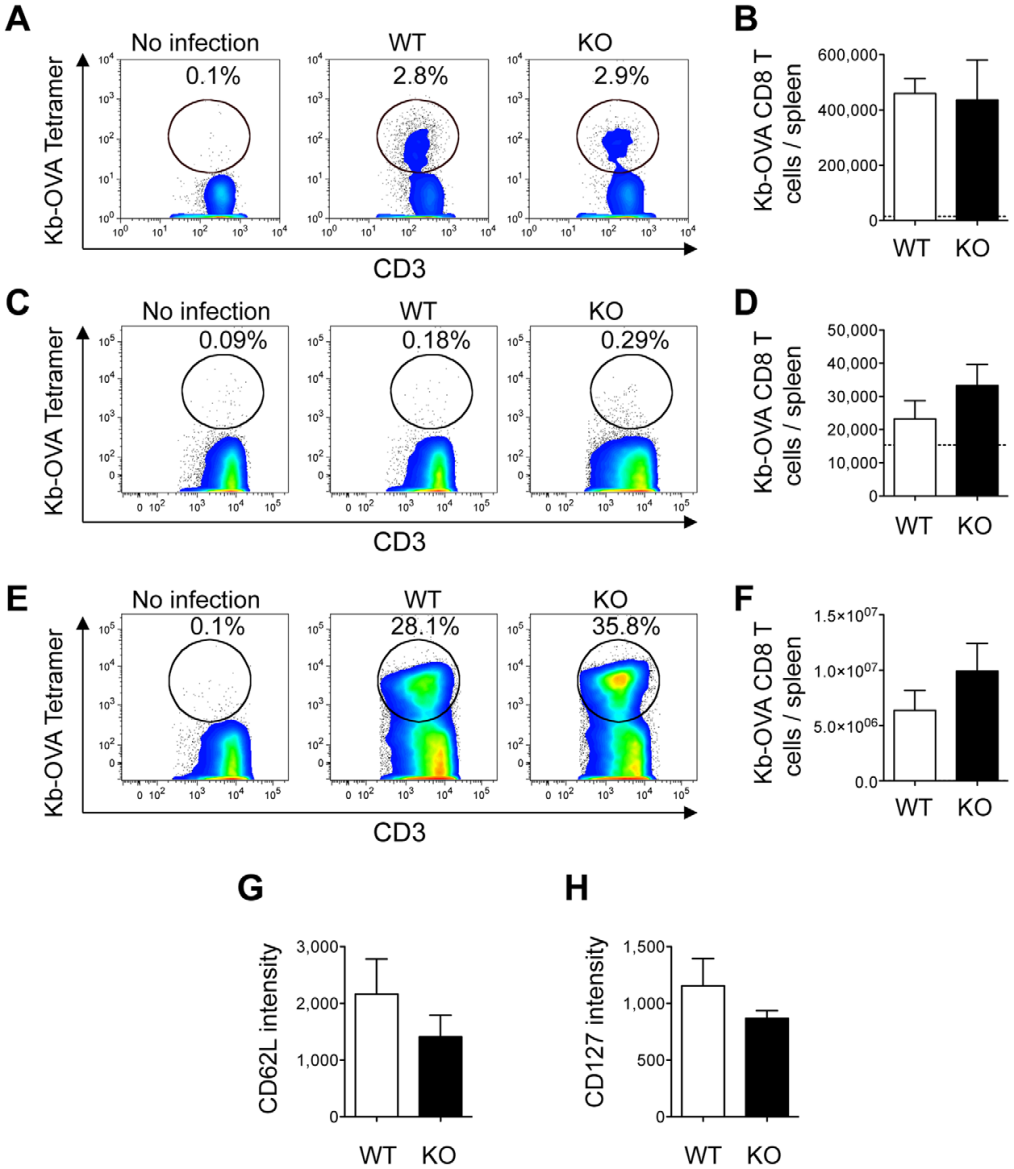
Normal effector and memory CD8+ T cell response to Listeria in KSR1-deficient mice. WT and KSR1-/- mice were infected with 2×10^3^ cfu of Lm-OVA, and presence of OVA epitope specific CD8+ T cells in the spleen was analyzed by flow cytometry 7 (A, B) and 30 (C, D, G, H) days after infection. 30 days after Lm-OVA infection, mice were challenged with 10^5^ cfu of Lm-OVA and presence of OVA epitope specific CD8+ T cells in the spleen was assessed 5 days later (E, F). A, C, E: Percentage of Db-GP33 Tetramer positive cells in CD8+ T cells. Plots are representative of two experiments with 3–8 mice per group. B, D, F: Number of Db-GP33 Tetramer+ CD8+ T cells per spleen. Dashed line represents average numbers in naive mice and mice infected with Lm. G, H: CD62L and CD127 expression in Db-GP33 Tetramer+ CD8+ T splenocytes 30 days after Lm-OVA infection. Data is presented as mean + SEM and is representative of two experiments with 3–8 mice per group. There were no significant differences between WT and KO mice (t-test).

We then tested the impact of KSR1-deficiency on the generation of CD8+ T cell memory in response to Listeria infection, as rapamycin-treatment also enhances CD8 T cell memory in this model [8]. To do so, we infected wild-type and KSR1-/- mice with recombinant *Listeria monocytogenes* expressing OVA (Lm-OVA), and analyzed the number and phenotype of OVA-specific CD8+ T cells by flow cytometry at different time points after infection. This dose of infection did not induce any lethality, and bacterial clearance was similar between wild-type and KSR1-deficient mice (data not shown). We did not detect any differences in the number or phenotype of effector (day 7, Figure 5A and 5B) or memory (day 30, Figure 5C, 5D, 5G and 5H) OVA-specific CD8+ T cells between wild-type and KSR1-/- mice. Furthermore, upon reinfection with Lm-OVA 30 days after the primary infection, OVA-specific CD8+ T cells expanded normally in KSR1-/- mice (Figure 5E and 5F), confirming that there is no defect in CD8+ T cell memory cells in KSR1-/- mice. Altogether, these data shows that despite the decreased mTOR activation in KSR1-deficient CD8+ T cells, KSR1-deficiency does not affect the generation and survival of memory CD8+ T cells.

## Discussion

mTOR plays a critical role in T cells to maintain normal T cell homeostasis under steady-state conditions, and influences their differentiation into distinct lineages [10]. The MAPK signaling pathway has been shown to participate in mTOR activation in different cell lines [13], [15], but whether it interacts with the mTOR pathway in T cells was unknown. Here we showed that the ERK signaling pathway was important for sustained mTORC1 activation in T cells following TCR engagement. However, while ERK signaling was impaired in KSR1-deficient mice, T cells did not exhibit any defect in the generation of Tregs or CD8+ memory T cells in KSR1-deficient mice. This data suggests that despite its role in mTORC1 activation *in vitro*, KSR1 is not required for T cell differentiation into regulatory and memory T cells *in vivo*.

Inhibition of the ERK signaling pathway with a MEK inhibitor, or the absence of KSR1 did not affect the early activation of mTOR, but only its sustained activation 48 hours after T cell activation. It is possible that different pathways are sequentially involved to activate mTOR. For instance, inhibition of the PI3K/AKT pathway in CD8+ T cells blocks mTORC1 activation as early as 2 hours following TCR engagement in the presence of costimulation [9]. Therefore, it is possible that the PI3K/AKT pathway is critical for early mTORC1 activation, whereas the ERK signaling pathway is required to maintain mTORC1 activation later on, but is not essential for the initial activation of mTORC1. Interestingly, we observed a biphasic activation of ERK after TCR engagement: a first wave of activation that decreased after 6 hrs of TCR stimulation, and a second wave of activation after 24 hrs to 48 hrs. It is therefore possible that only the second wave of ERK activation participates in mTOR activation. The transient down-regulation of ERK phosphorylation might be a consequence of TCR down-regulation, or to the activity of MAPK phosphatases, as it has been suggested that different MAPK phosphatases might intervene at different time and location to control the duration and intensity of MAPK phosphorylation [27].

mTOR inhibition is thought to favor the development of regulatory CD4+ T cells and memory CD8+ T cells through its action on metabolism [10]. Indeed, both CD4+ Tregs and CD8+ memory T cells have much lower metabolic demands than conventional effector T cells, and for example depend more on fatty acid oxidation, a process regulated by mTOR and AKT [8], [28]. mTOR controls metabolic pathways through its regulation of different transcription factors, such as HIF1α, MYC and SREBP [10]. mTOR also controls the expression and/or activity of different transcription factors associated with T cell fates, such as STAT transcription factors and FoxP3 for CD4+ T cells [3], [5], and eomesodermin and T-bet for effector and memory CD8+ T cells [9]. Considering that mTOR activity is reduced in KSR1-deficient T cells, we expect that KSR1 deficiency also has an impact on these transcription factors, as well as mTOR-regulated metabolic pathways but this remains to be tested.

mTOR inhibition in CD4+ T cells leads to impaired differentiation into Th1, Th2 and Th17 T cells [3], [5], [6]. Similarly, rapamycin-treated CD8+ T cells have defective effector functions [9]. Despite the decreased mTOR activation in KSR1-deficient T cells, KSR1-/- T cells can differentiate normally into Th1 and Th2 cells *in vitro* [17]. Our data also suggest that KSR1-/- CD8+ T cells can differentiate into efficient effector T cells *in vivo*, as they were able to expand normally after *Listeria monocytogenes* and LCMV infections, and as KSR1-/- mice do not have any defect in Lm clearance. Similarly, in contrast to rapamycin-treated mice [7], [8], KSR1-/- mice generate normal memory CD8+ T cell responses, even if KSR1-deficient T cells expressed higher levels of CD127 (IL-7Rα, essential for the maintenance of memory T cells [24]) after activation *in vitro*. The absence of phenotype in KSR1-/- mice might come from the fact that early mTORC1 activation is normal in KSR1-/- T cells, and only sustained mTORC1 activation is impaired. However, this cannot explain the normal memory CD8+ T cell development in KSR1-/- mice, as sustained mTORC1 activation is thought to be required for the acquisition of effector function by CD8+ T cells at the expense of memory CD8+ T cell generation [9]. In contrast to rapamycin-treated cells, however, mTORC1 activation was not completely abolished in KSR1-/- T cells, but only reduced by 30 to 40%. The remaining mTORC1 activity might be enough to allow for normal effector versus memory CD8+ T cell differentiation, as well as normal Th1 and Th2 CD4+ T cell differentiation.

Blocking the mTOR pathway has been shown to increase the thymic development of natural Tregs [25]. However, KSR1-deficient mice displayed normal numbers or Tregs in the thymus and in the spleen, showing that KSR1 is not required for natural Treg development despite its effect on mTORC1 activation. This might be due to the residual mTORC1 activity in KSR1-/- CD4+ T cells. Alternatively, as for induced Tregs [3], signals through mTORC2 might be sufficient to control natural Treg development when mTORC1 activity is decreased. Indeed, both mTORC1 and mTORC2 absence are required to increase the differentiation of induced Tregs from naïve CD4+ T cells [3].

Another explanation for the absence of phenotype in T cell development in KSR1-/- mice, and the mild effect of KSR1 deficiency on the mTOR pathway, is that there might be some compensatory effects from the KSR1 ortholog KSR2, as KSR2 can also facilitate the activation of the MAPK pathway [20]. KSR2 might therefore be sufficient to activate the mTOR pathway in the absence of KSR1, through the activation of the MAPK pathway, and allow for the normal generation of memory and regulatory T cells in KSR1-deficient mice. However, if KSR2 has any effect on the mTOR pathway, this effect might not be positive but rather inhibitory, as KSR2 also activates the AMPK pathway [29], [30], which would lead to mTOR inhibition. Furthermore, it is unlikely that KSR2 compensates for KSR1 in T cells, as we were unable to detect KSR2 expression in T cells and thymocytes by western blot or RT-PCR (data not shown). In normal conditions, KSR2 protein expression has only been reported in the brain and adipose tissue [29]; KSR2 might also be expressed at lower levels in liver and skeletal muscles, as KSR2 mRNA has been detected in these tissues [29], but never in T cells.

Overall, this work demonstrates that KSR1 deficiency has little to no effect on the generation of CD4+ regulatory and CD8+ memory T cells *in vivo*, despite its role in mTOR activation *in vitro*; we could not detect any difference in memory CD8+ T cell response or Treg development *in vivo* between KSR1-deficient mice and wild-type mice at homeostasis and in different infectious models. This also suggests that only strong perturbations of the mTOR pathway lead to altered Treg and memory T cell generation, as the decrease in mTOR activation in KSR1-deficient T cells was not sufficient to alter Treg and memory T cell generation *in vivo*, in contrast to what has been published for mTOR-deficient and rapamycin-treated mice. However, it is possible that blocking KSR1 and ERK signaling might affect T cell differentiation under suboptimal conditions. Antigen dose has been shown to affect effector versus regulatory CD4+ T cell differentiation by modulating the activation of mTORC1, with low antigen dose inducing stronger mTOR activation associated to increased Treg and decreased effector differentiation [31]. Therefore, KSR1 and ERK signaling might be required for correct T cell differentiation when limited amount of antigen are present.

## Materials and Methods

### Ethics statement

Care and use of mice were conducted in accordance with protocols approved by the Washington University Animal Studies Committee (ASC), in compliance with the Animal Welfare Act. All mice were housed under specific pathogen-free conditions in the Washington University animal facilities. At the end of experiments, animals were sacrificed by carbon dioxide overdose.

### Mice

KSR1 knockout mice on a C57BL/6J background were previously described [17]. Age and gender-matched C57BL/6J controls were used for all experiments.

### Cell culture and antibodies

T cells isolated from C57BL/6J and KSR1-deficient mice T cells were cultured in IMDM supplemented with 10% FBS, 2 mM glutamine, nonessential amino acids, sodium pyruvate, 2-ME, penicillin, and streptomycin. UO126 inhibitor was purchased from Sigma-Aldrich. TCR stimulation was performed with anti-mouse CD3 (2C11) and anti-mouse CD28 (37.51). Anti-pT202/T204-ERK, anti-pT421/S424-S6K and anti-pT389-S6K were from Cell Signaling Technology. Anti-mouse CD3-Pacific Blue (17A2), CD8-APC or -APC-Cy7 (53–6.7), and CD62L-Pacific Blue (MEL-14) were from Biolegend. Anti-mouse CD3-APC (145.2C11), CD4-FITC or -PECy7 (RM4-5), CD25-PE (PC61), and CD127-PECy7 (SB/199) were from BD Biosciences. Anti-mouse CD44-FITC (IM7) and FoxP3-Alexa Fluor 647 (FJK-16s) were from eBioscience. Anti-rabbit-PE and anti-mouse-PE secondary antibodies were from Jackson ImmunoResearch Laboratories. PE-H2-K^b^/OVA_257–264_ (Kb-OVA) MHC-peptide tetramer was generated from single chain trimer as previously described [32], and PE-H2-D^b^/GP33_33–41_ (Db-GP33) MHC-peptide tetramer was from Beckman Coulter.

### Flow cytometry

Single-cell suspensions were generated from spleens or thymuses. Detection of phosphorylated proteins was done as described previously [33]. Briefly, cells were stimulated with anti-CD3 + anti-CD28 (5 µg/mL each) for 2 hrs to 48 hrs. Cells were fixed with 0.5% paraformaldehyde and permeabilized with 95% methanol. Cells were first stained with anti-pERK1/2, anti-pT421/S424-S6K or anti-pT389-S6K followed by secondary antibody, and then stained with anti-CD4 and anti-CD8 antibodies. For FoxP3 intracellular staining, cells were first stained for CD3, CD4, CD25 and CD8, then fixed and permeabilized using Cytofix/Cytoperm (BD Biosciences), and then stained for FoxP3. FACS analyses were performed on a FACS Calibur or a FACS Canto II (BD Biosciences).

### Western blot

Mouse splenocytes were activated for 48 hrs with anti-CD3 + anti-CD28 antibodies. T cells were then enriched by Dynal negative selection kit (>95% purity) at 4°C.

The pellet was re-suspended in ice-cold lysis buffer (0.1 M Tris base, 140 mM NaCl, 1 mM EDTA, 1% NP-40, 1 mM phenylmethylsulfonyl fluoride, 1 mM sodium orthovanadate, and 50 mM sodium fluoride). After centrifugation, proteins from cell lysates were resolved by sodium dodecyl sulfate-polyacrylamide gel electrophoresis (SDS-PAGE) and analyzed by immunoblotting with the indicated primary antibodies followed by incubation anti-rabbit or anti-mouse IgG coupled to horseradish peroxidase. ECL western blotting substrate (Pierce) was used for detection. Band intensity for quantification was measured using ImageJ.

### Infections

6- to 8-week-old mice were infected by intraperitoneal injection with 2×10^5^ pfu LCMV-Armstrong, or 2×10^3^ cfu of recombinant *Listeria monocytogenes* (Lm) expressing OVA (Lm-OVA) [34]. 30 days after infection with Lm-OVA, mice were challenged by intraperitoneal injection of 10^5^ cfu of Lm-OVA. For rapamycin treatment, mice received daily intraperitoneal injections of rapamycin from day -1 before infection with LCMV-Armstrong to day 30 post-infection. The daily dose of rapamycin was 75 µg/kg from day -1 to day 7, and 600 µg/kg from day 8 to day 30, as previously described [7].

### Statistical analysis

GraphPad Prism was used for graphing and statistical analysis.

## Supporting Information

Figure S1 **Decreased mTOR activity in MEK-inhibited and KSR1-deficient T cells.** WT, UO126-treated WT, and KSR1-deficient splenocytes cells were cultured for 48 hrs with or without anti-CD3 + anti-CD28 stimulation (5 µg/mL each). T cells were purified, lysed and resolved on SDS-PAGE gels. A: Lysates were blotted with the indicated antibodies. B: Blots were quantified with Image J. white bars: WT, grey bars: WT+UO126, black bars: KSR1-/-; NT: not tested.
(TIF)
