# Supplementary figures and images for: Kinase Suppressor of Ras 1 Is Not Required for the Generation of Regulatory and Memory T Cells
Source: PLoS One. 2013 Feb 19;8(2):e57137. doi: 10.1371/journal.pone.0057137 (PMC3576348; doi:10.1371/journal.pone.0057137)

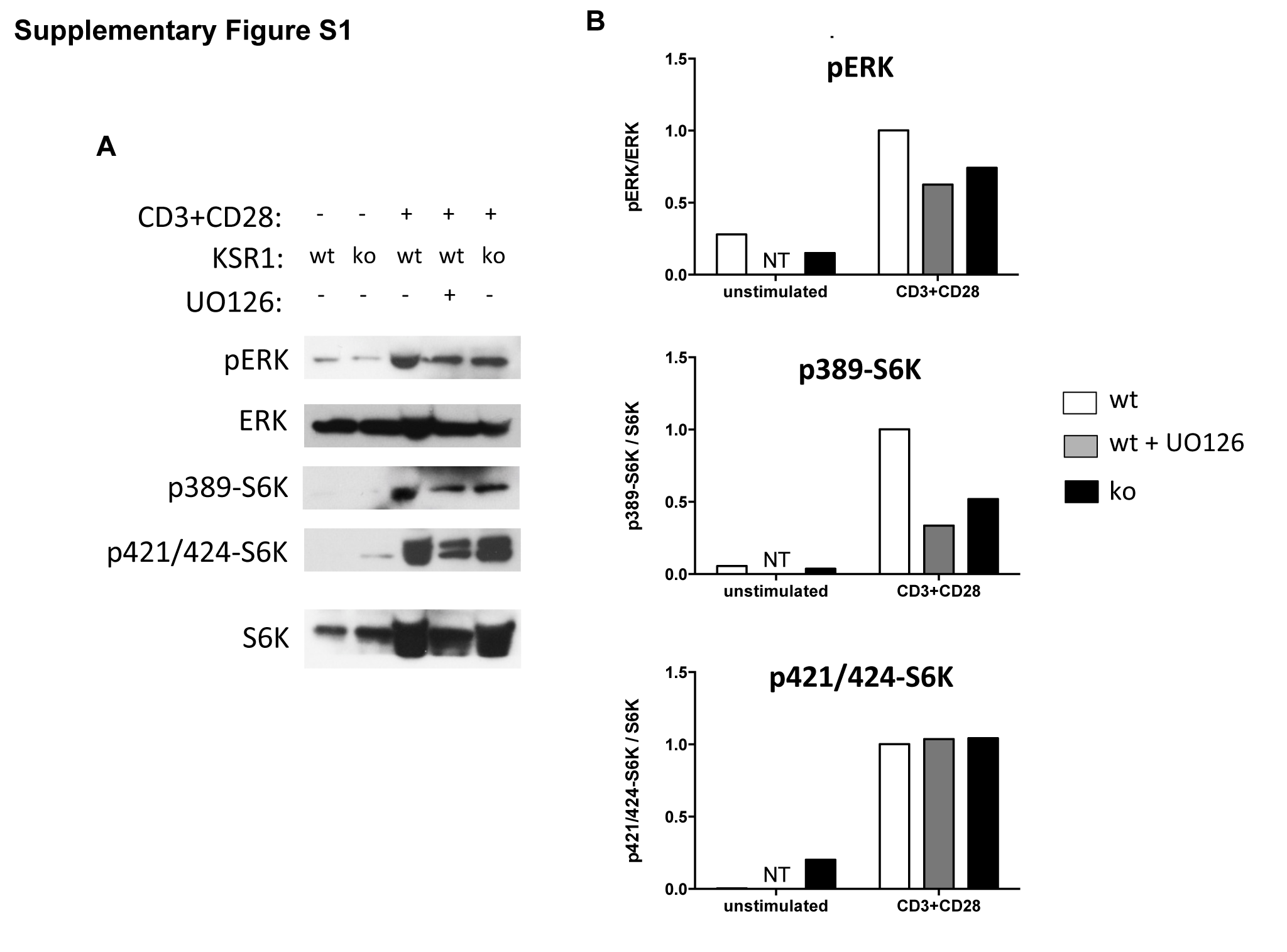

Supplement: Figure S1 — Decreased mTOR activity in MEK-inhibited and KSR1-deficient T cells. WT, UO126-treated WT, and KSR1-deficient splenocytes cells were cultured for 48 hrs with or without anti-CD3 + anti-CD28 stimulation (5 µg/mL each). T cells were purified, lysed and resolved on SDS-PAGE gels. A: Lysates were blotted with the indicated antibodies. B: Blots were quantified with Image J. white bars: WT, grey bars: WT+UO126, black bars: KSR1-/-; NT: not tested. (TIF) [file pone.0057137.s001.tif]
